# Exploring Multidimensional Risk Factors Associated with Local Adverse Reactions to Subcutaneous Immunoglobulin Therapy: Insights from a Nationwide Multicenter Study

**DOI:** 10.3390/biomedicines13081991

**Published:** 2025-08-15

**Authors:** Sandra Martínez Mercader, Victor Garcia-Bustos, Pedro Moral Moral, Carmen Martínez Buenaventura, Elisa Escudero Vergara, María Carmen Montaner Bosch, Héctor Balastegui-Martín, Sonia Galindo Maycas, Miriam González Amores, Noemí Gimenez Sanz, Marian Escobar Palazón, María Moreno Mulet, Ignacio Campanero Carrasco, Alicia López, Carlos Daniel Hernández Ruiz, Laura Ruiz-López, Rocío Guzmán Guzmán, Marta Dafne Cabañero-Navalon

**Affiliations:** 1 Primary Immunodeficiencies Unit, Department of Internal Medicine, University and Polytechnic Hospital La Fe, 46026 Valencia, Spaincabanero_marnav@gva.es (M.D.C.-N.); 2 Severe Infection Research Group, Health Research Institute La Fe, 46026 Valencia, Spain; 3 Research Group of Chronic Diseases and HIV Infection, Health Research Institute La Fe, 46026 Valencia, Spain; 4 Pediatric Infectious Diseases and Immunodeficiencies Unit, Vall d’Hebron University Hospital, 08035 Barcelona, Spain; 5 Infection and Immunity in Pediatric Patients Research Group, Vall d’Hebron Institut de Recerca, 08035 Barcelona, Spain; 6 Area of Immunology—Multidisciplinary Day Hospital, Gregorio Marañón General University Hospital, 28007 Madrid, Spain; 7 Immunology Day Hospital Unit, Reina Sofía University Hospital, 14004 Córdoba, Spain; 8 Onco-Hematology Day Hospital, La Paz University Hospital, 28046 Madrid, Spain; 9 Internal Medicine Day Hospital, Doctor Negrín University Hospital of Gran Canaria, 35010 Gran Canaria, Spain; 10 Clinical Immunology and Primary Immunodeficiencies Unit, Pediatric Allergy and Clinical Immunology Department, Sant Joan de Déu Hospital, 08950 Barcelona, Spain; 11 Day Hospital, Son Espases University Hospital, 07120 Palma de Mallorca, Spain

**Keywords:** common variable immunodeficiency (CVID), immunoglobulin replacement therapy (IgRT), subcutaneous immunoglobulin (SCIg), intravenous immunoglobulin (IVIg), real-world evidence

## Abstract

**Background/Objectives**: Subcutaneous immunoglobulin (SCIg) is a well-established alternative to intravenous immunoglobulin (IVIg) in patients with primary (PID) and secondary immunodeficiency (SID), with demonstrated benefits in safety and quality of life. However, its implementation remains limited in parts of Southern Europe, partly due to frequent local adverse reactions (LARs), which, despite being mild, can affect adherence and clinician confidence. This study aimed to identify clinical, anatomical, psychosocial, and geographical factors associated with LARs and to develop an exploratory model for individualized risk estimation. **Methods**: We conducted a retrospective, multicenter observational study in eight Spanish hospitals using data from the GEIE Registry. Patients aged ≥14 years with PID or SID receiving SCIg for ≥1 month were included. Demographic, clinical, anatomical, and psychosocial variables were collected. A multivariable logistic regression model was built to identify independent predictors of LARs and internally validated using bootstrap resampling (500 iterations). A nomogram was constructed for personalized risk prediction. **Results**: Among 223 included patients, 73.1% reported LARs, primarily swelling, pruritus, and rash. Independent predictors included smaller abdominal perimeter (OR 0.955, *p* < 0.001), history of skin disease (OR 2.75, *p* = 0.044), greater distance to hospital (OR 1.01, *p* = 0.050), and absence of anxiety (OR 0.089, *p* = 0.001). Model discrimination was good (AUC 0.801), with minimal optimism after internal validation (validated AUC 0.788). **Conclusions**: LARs are common among patients receiving SCIg and could be influenced by anatomical, dermatological, psychological, and geographical factors. This exploratory multicenter study underscores the clinical relevance of these factors and may guide more personalized and safer use of SCIg.

## 1. Introduction

Immunoglobulin replacement therapy (IgRT), administered either intravenously (IVIg) or subcutaneously (SCIg), is the standard of care for patients with primary (PID) and secondary immunodeficiency (SID), conditions characterized by impaired antibody production and increased susceptibility to infection [1,2]. While both routes are equally effective in preventing infections and preserving organ function, SCIg offers important advantages: more stable IgG serum levels, fewer systemic adverse events, enhanced quality of life, and greater patient autonomy [3,4,5].

However, access to and uptake of SCIg vary considerably across Europe, reflecting differences in national health policies, clinical practice patterns, and healthcare infrastructure. According to the European Immunoglobulin Map [6], SCIg use was already widespread in several Northern and Central European countries by 2014, where it is often used as first-line treatment and frequently administered at home. In Southern Europe, however, implementation has remained limited. In Spain, although SCIg has been available since 2004, its utilization remained below 15%, with considerable variability across centers [6,7]. Recent national data suggest a modest increase in adoption; however, its use continues to be limited, particularly in non-specialized settings lacking dedicated immunodeficiency care teams [8,9].

Several barriers may explain this persistent underuse. These include a lack of familiarity with SCIg protocols among non-specialized providers, limited availability of trained nursing staff to deliver patient education, and the logistic demands of self-administration, which requires infusion devices, consumables, and coordination [10,11,12]. One of the most frequently cited concerns, however, is the high incidence of local adverse reactions (LARs), such as erythema, pruritus, or swelling at the injection site [13]. Although these reactions are usually mild and self-limiting, their frequency and variability may negatively impact patient adherence and provoke hesitation among prescribing clinicians. This reluctance persists despite SCIg’s more favorable systemic safety profile compared to IVIg and its consistent association with better long-term tolerability and patient-reported outcomes [3,4].

In this context, a better understanding of the factors associated with LARs could enhance clinical decision-making and guide more effective patient education. This study aimed to examine clinical, anatomical, psychosocial, geographical, and treatment-related variables linked to LARs in a real-world cohort of patients with PID and SID across Spain. Through an exploratory analysis, we sought to identify relevant patterns that may inform individualized risk assessment and contribute to safer, more widespread adoption of SCIg therapy.

## 2. Materials and Methods

### 2.1. Study Design and Ethical Statement

A multicenter, retrospective observational study was conducted across eight Spanish hospitals between November 2023 and August 2024. This study included patients aged 14 years or older with a confirmed diagnosis of either PID or SID, who had received SCIg for at least one month. The cohort was extracted from the GEIE Spanish Registry, an initiative coordinated by the Immunodeficiency Nursing Group of the Spanish Society of Immunology (SEI), aiming to evaluate real-world outcomes of immunoglobulin therapy in patients with immunodeficiencies [9].

The protocol of the GEIE Spanish Registry was approved by the institutional ethics committees of all participating centers, with corresponding registry codes. All procedures adhered to the principles of the Declaration of Helsinki, Spanish regulations on observational studies, and the STROBE guidelines. Patient anonymity and data confidentiality were strictly maintained.

### 2.2. Patient Characterization and Variable Definition

The methodology for data collection followed the same standardized approach as previously described by Martínez-Mercader et al. (2025) [9], using the GEIE Spanish Registry framework. This registry prospectively compiles clinical, sociodemographic, and psychosocial information on patients with immunodeficiencies undergoing IgRT in routine clinical practice. Data were retrospectively retrieved from electronic medical records and complemented through structured interviews conducted by trained nurses specialized in the care of immunodeficiency patients.

Particular emphasis was placed on treatment-related variables, including the type of SCIg formulation, weekly dosage, total infusion volume, administration frequency, infusion duration, number and location of injection sites, and technical aspects such as needle gauge and length. Additional variables included the use of anticoagulant or antiplatelet therapy, as well as the patient’s abdominal perimeter and the number of nurse-led training sessions required for self-administration. Adverse reactions were systematically documented based on patient-reported experience and classified as local (LARs) (e.g., erythema, rash, pruritus, swelling, leakage, hematoma, nodules, necrosis, ulceration) or systemic (e.g., headache, fever, asthenia, myalgia, arthralgia, generalized rash, wear-off phenomena).

Demographic and clinical data encompassed age, sex, diagnosis (PID or SID), and subtype in the case of PID. Comorbidities of interest included dermatological conditions, autoimmune cytopenias, benign lymphoproliferative disorders, chronic lung disease, enteropathy, liver disease, and history of malignancies, solid organ transplantation, or hematopoietic stem cell transplantation.

Information on patients’ socioeconomic status included the distance from their residence to the referral hospital and their current employment situation. The Gijón Scale [14] was used to assess familial and social circumstances, including household composition, income level, housing quality, social interactions, and support networks. In addition, participants completed the EuroQol-5D-3L questionnaire [15], which evaluates five key dimensions—mobility, self-care, usual activities, pain or discomfort, and anxiety or depression—and incorporates a visual analog scale to quantify overall perceived health on a scale from 0 to 100.

### 2.3. Statistics

The statistical analysis was performed with R version 4.4.1 software. Descriptive statistics were used to summarize the demographic, clinical, treatment-related, and psychosocial characteristics of the study population. Continuous variables were expressed as means with standard deviations or medians with interquartile ranges, as appropriate, and compared using Student’s *t*-test or the Mann–Whitney U test based on distribution. Categorical variables were summarized as counts and percentages and compared using the Chi-square or Fisher’s exact tests, as appropriate. A two-sided *p*-value < 0.05 was considered statistically significant.

To identify independent risk factors for LARs, a multivariable logistic regression analysis was conducted. Prior to model fitting, multicollinearity among candidate predictors was assessed using the Variance Inflation Factor (VIF). Variables with VIF values exceeding 5 were excluded to mitigate collinearity-related distortions in coefficient estimates. In parallel, variable selection was refined using elastic net regularization, which integrates both L1 (lasso) and L2 (ridge) penalties to optimize model performance and prevent overfitting. Final variable inclusion was determined based on a combination of prior scientific evidence, biological plausibility, elastic-net-derived contribution, and acceptable multicollinearity (VIF < 5). Model discrimination was evaluated using the area under the receiver operating characteristic curve (AUC), and calibration was visually assessed by plotting predicted versus observed probabilities. Additionally, the model was internally validated using the bootstrap resampling technique using 500 bootstrap iterations. Iterations with invalid values (NA) were excluded. In each iteration, the model was refitted on a bootstrap sample and evaluated on both the bootstrap and original datasets to estimate optimism. The average optimism was subtracted from the original AUC to obtain the optimism-corrected AUC, reflecting the model’s validated discriminative performance. Finally, a nomogram was constructed from the final logistic regression model to enable individualized, visually guided risk estimation of LARs.

## 3. Results

### 3.1. Demographics

The study included 223 participants, with women comprising the majority (61.4%, *n* = 137) and men accounting for 38.6% (*n* = 86). The mean age at inclusion was 47.1 years (SD = 18.6). Of the total cohort, 65.0% (*n* = 145) were diagnosed with primary immunodeficiency (PID), while 35.0% (*n* = 78) had secondary immunodeficiency (SID). Among those with PID, the most frequent diagnosis was common variable immunodeficiency (CVID) (39.8%, *n* = 88), followed by IgG subclass deficiency (6.3%, *n* = 14), combined IgA and IgG subclass deficiency (5.9%, *n* = 13), and syndromic immunodeficiencies (5.4%, *n* = 12). In the SID group, leading causes included immunosuppressive or chemotherapeutic treatment for lymphoma (33.3%, *n* = 26), myeloma (24.4%, *n* = 19), autoimmune disorders (12.8%, *n* = 10), and leukemia (6.4%, *n* = 5). Most patients (98.3%) performed SCIg administration at home after appropriate training, while only a small minority received it in a healthcare facility. Further data on the patient comorbidities, therapeutic regime, socioeconomic status, and health perception of the included patients can be consulted elsewhere (Martínez-Mercader et al., 2025 [9]).

### 3.2. Global Adverse Effects of SCIg

Of the total of 223 patients, 166 (74.43%) reported at least one local or systemic adverse effect related to SCIg therapy. Among these, LARs were the most frequently reported, occurring in 163 patients (98.19%), whereas systemic adverse effects were documented in 67 patients (40.36%). Notably, only three patients (1.81%) experienced systemic reactions in the absence of any local adverse events.

On the one hand, among specific systemic adverse effects related to SCIg therapy, headache was the most frequently reported, occurring in 25.9% of patients. Other systemic symptoms included asthenia (17.47%), wear-off effects (24.63%), arthromyalgia (13.86%), and fever (7.83%). Systemic rash was infrequent, reported in only 3.61% of cases.

On the other hand, among patients reporting LARs to SCIg, swelling and pruritus were the most frequently observed reactions, affecting 70.48% and 65.45% of patients, respectively. Rash was also common, reported by 65.06% of patients, followed by pain at the injection site (31.93%). Less frequent local reactions included hematoma and leakage (both 10.84%), nodules (7.45%), necrosis (4.82%), and ulceration (2.41%) (Table 1).

### 3.3. Local Adverse Effects of SCIg: Patient Profiles by Local Tolerability

Among the total of 223 included patients, 163 (73.1%) experienced at least one LAR, while 60 (26.9%) reported no such events. A global overview of the patient profiles by local tolerability is presented in Table 2. The mean age of the overall cohort was 48.1 years (SD 18.3), with no significant age difference between those with and without local reactions (46.0 vs. 50.2 years, *p* = 0.131). The sex distribution was balanced across groups (38.0% vs. 40.0% male, *p* = 0.877).

The majority of patients had primary immunodeficiency (PID), with no significant differences in distribution between groups (63.2% in the local reaction group vs. 70.0% in controls, *p* = 0.429). Secondary immunodeficiency (SID) accounted for 36.8% and 30.0% of cases, respectively. Comorbid cytopenia (10.2% vs. 15.0%, *p* = 0.338), immune thrombocytopenia (5.5% vs. 10.0%, *p* = 0.239), anticoagulant use (6.8% vs. 8.3%, *p* = 0.770), and history of hematopoietic (10.4% vs. 6.8%, *p* = 0.604) or solid organ transplantation (4.5% vs. 1.7%, *p* = 0.451) were similarly distributed across groups. Although differences were not statistically significant, an exploratory analysis showed that patients with solid organ transplantation more frequently used the thigh for infusion compared to non-transplanted patients (25.0% vs. 13.5%). Among patients with hematopoietic stem cell transplantation, use of non-abdominal sites was also slightly higher, such as thighs (13.3% vs. 8.8%) and combinations including arms. Due to the small sample size (8 with SOT and 21 with HSCT), these findings are descriptive and should be interpreted with caution.

Regarding treatment characteristics, the mean weekly SCIg dose was comparable between groups (8.02 g vs. 8.10 g, *p* = 0.835), as was total infusion volume (155.9 mL vs. 159.8 mL, *p* = 0.888). Administration frequency showed a non-significant trend (*p* = 0.051), with weekly regimens being the most common in both groups (39.9% vs. 50.0%). There were no significant differences in the occurrence of LARs between patients receiving 10% and those receiving 20% immunoglobulin formulations (*p* = 0.131), despite the intrinsic differences in infusion volumes dictated by concentration and administration method. In our cohort, all 10% preparations were administered with recombinant human hyaluronidase, enabling the delivery of substantially larger volumes at a limited number of sites, whereas 20% preparations—given without hyaluronidase—required smaller volumes and shorter infusion times. The number of simultaneous injection sites was comparable between groups (1.84 vs. 1.93, *p* = 0.124). However, patients who experienced LARs more frequently received infusions with longer needles (e.g., 12 mm: 11.7% vs. 1.7%, *p* = 0.023).

Injection site differed significantly: while abdominal administration predominated overall, it was less frequent among patients with LARs (54.0% vs. 81.7%, *p* = 0.002), who more often used alternate sites. Infusion time was shorter among those experiencing reactions (60.7 vs. 80.8 min, *p* = 0.047), and their abdominal perimeter was significantly smaller (87.3 vs. 94.9 cm, *p* < 0.001). The number of patient education sessions received also differed, with more patients in the adverse effect group attending more than three sessions (43.6% vs. 28.3%, *p* = 0.045).

Geographical and socioeconomic characteristics showed notable differences. Patients with local adverse effects lived farther from their reference hospital (mean 35.0 vs. 20.8 km, *p* = 0.005). Lower socioeconomic status was more prevalent in this group, including a higher proportion of patients with non-contributory pensions (15.0% vs. 1.9%) or no income (19.1% vs. 6.7%) (*p* < 0.001).

In terms of psychosocial variables, there were no significant differences in family setting, labor status, or social relationships. However, emotional and physical well-being differed markedly. Patients reporting no anxiety or depression were more prevalent among controls (96.6% vs. 70.6%), while moderate or severe anxiety was more common in those with adverse effects (*p* < 0.001). Similarly, moderate discomfort or pain was significantly more frequent in the affected group (39.1% vs. 13.8%, *p* < 0.001), despite similar levels of mobility and independence.

### 3.4. Multivariable Prediction Model for Local Adverse Effect of SCIg

**Figure 1:**
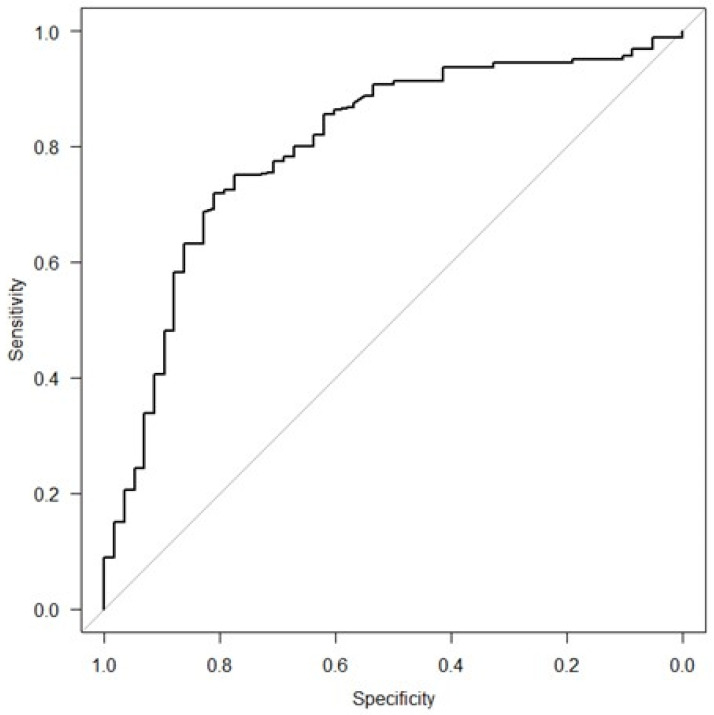
Area under the receiver operating characteristic curve (AUC) of the multivariable exploratory model for predicting local adverse effects in patients receiving SCIg (AUC of 0.801).

A multivariable logistic regression model was created to assess the independent risk factors for the referral of LARs in patients receiving SCIg. The model demonstrated good discriminative performance, with an area under the receiver operating characteristic curve (AUC) of 0.801 (95% CI: 0.733–0.869) (Figure 1). Physical, dermatological, geographic, and psychological factors were relevant to SCIg tolerability. Larger abdominal perimeter was associated with lower risk (OR 0.955, *p* < 0.001), while a history of skin disease significantly increased risk (OR 2.750, *p* = 0.044). Distance to hospital in km showed a marginal association (OR 1.010, *p* = 0.050). The absence of self-reported anxiety or depression was strongly protective against LARs (OR 0.089, *p* = 0.001), indicating that patients reporting anxiety or depression had a markedly higher risk of developing LARs. (Table 3). The internal validation by means of bootstrap resampling technique on 500 iteration samples yielded a mean optimism of 0.012, indicating minimal overestimation of model performance. After correcting for optimism, the validated AUC was 0.788, reflecting good discriminative ability with a low risk of overfitting.

A nomogram was developed to graphically represent the multivariable logistic regression model, incorporating statistically significant predictors of local adverse reactions to SCIg: absence of self-reported anxiety or depression, previous skin disease, hospital distance (km), and abdominal perimeter (cm). The absence of anxiety or depression showed a protective effect, while previous skin disease was associated with increased risk. Continuous variables were modeled as linear terms.

**Figure 2:**
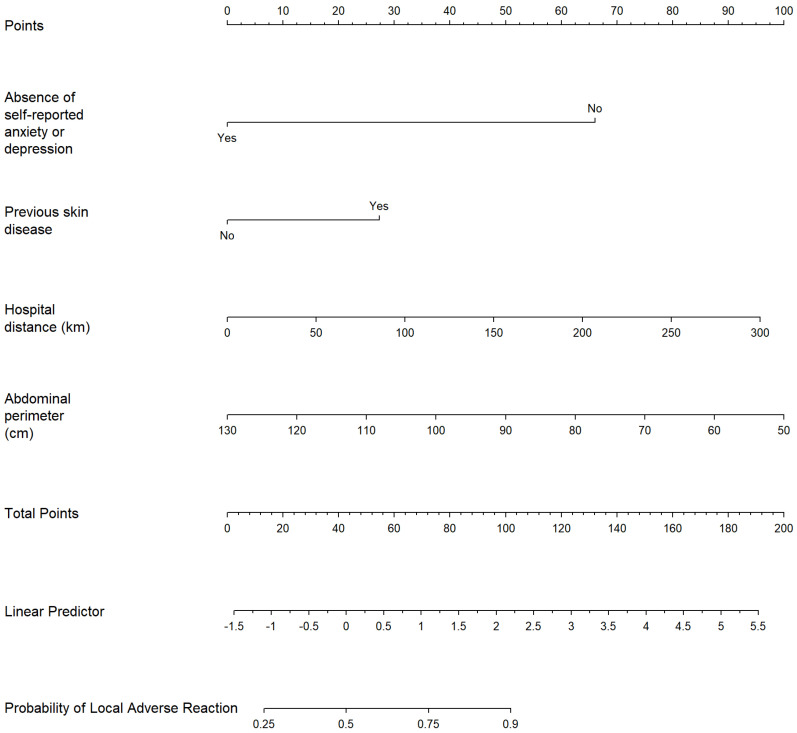
Nomogram representing the predictive probability of reporting local adverse reactions to subcutaneous immunoglobulin therapy.

Each predictor was assigned a weighted score based on its regression coefficient, and the total score was translated into an estimated probability of experiencing a local adverse reaction (Figure 2).

Each predictor in the multivariable logistic regression model is aligned with a corresponding point scale at the top of the nomogram. To estimate the individual risk, locate the patient’s value for each variable (absence of self-reported anxiety or depression, previous skin disease, hospital distance in kilometers, and abdominal perimeter in centimeters), draw a vertical line to the “Points” axis to determine the score for each predictor, and sum the total points. The total score is then mapped to the lower scales to obtain the linear predictor and the corresponding probability of a local adverse reaction.

## 4. Discussion

In this multicenter, nursing-led study, we identified risk factors for LARs to SCIg therapy in a large real-world cohort of patients with primary and secondary immunodeficiencies across Spain. Our findings highlight the multifactorial nature of SCIg tolerability, with four independent variables retained in the final exploratory predictive model: (i) smaller abdominal perimeter, associated with increased LAR risk; (ii) personal history of skin disease, which significantly elevated susceptibility; (iii) greater distance from the patient’s home to the treating hospital, potentially reflecting reduced access to early education or follow-up; and (iv) presence of anxiety or depression, with the absence of self-reported symptoms showing a strong protective effect. The model demonstrated robust discriminative performance, even after internal validation through bootstrap resampling technique, underscoring the relevance of integrating anatomical, dermatological, geographical, and psychological parameters related to SCIg local tolerability.

IgRT use has progressively expanded in Spanish hospitals, supported by established evidence in PID [1] and increasing recognition of its role in SID, particularly in the context of novel therapies for hematological malignancies associated with prolonged iatrogenic hypogammaglobulinemia [16]. Despite demonstrated benefits of SCIg over IVIg—including improved quality of life, greater patient autonomy, lower overall healthcare costs, and reduced systemic adverse events—its adoption remains limited in Southern European countries [6,8]. Beyond the absence of standardized clinical guidelines, protocols, and logistical support in both secondary and tertiary care settings, limited awareness among clinicians managing PID and SID patients regarding LARs may represent a key barrier to the routine implementation of SCIg in this setting [9]. Between 30% and nearly 80% of patients receiving SCIg may experience LARs, regardless of severity, according to multiple series [9,17,18,19]. These reactions are typically mild and transient, often attributable to the subcutaneous administration of exogenous fluid volumes [20,21,22].

Factors predisposing to the development of LARs are multifactorial, encompassing both administration-related and patient-dependent variables. Administration-related determinants have been studied, yet some remain controversial and may vary depending on the specific SCIg formulation used [5,17,22,23,24,25]. Overall, slower infusion rates and the distribution of the dose across multiple infusion sites have been consistently associated with lower LAR rates, as also observed in our cohort [17,22].

In contrast, patient-related factors—such as comorbidities, psychosocial characteristics, and socioeconomic or demographic variables—remain poorly defined and insufficiently characterized in the current literature. One study reported lower LAR rates in patients with lower body mass index (BMI) [26], whereas in our cohort, a greater abdominal perimeter was notably associated with a protective effect. This finding may be attributable to the inclusion of exclusively pediatric patients, who generally require lower total infusion volumes. In addition, as a result of these findings, we hypothesize that standardized assessment of the subcutaneous tissue—either through BMI or direct measurement—could refine risk stratification and guide injection site selection. In this regard, the independent association between smaller abdominal perimeter and an increased risk of local adverse reactions suggests that avoiding or rotating injection sites—particularly in patients with reduced subcutaneous abdominal tissue—may be a useful and potentially modifiable strategy in clinical practice.

In line with our results, age was not associated with an increased risk of LARs, supporting the safety of SCIg administration across older patient populations [25]. Notably, prior use of therapeutic anticoagulation or antiplatelet agents, which could be considered potential barriers to SCIg administration by some clinicians, was not associated with an increased risk of LARs, either in univariate or multivariate analysis. This finding aligns with previous evidence [27], reinforcing the safety of SCIg in patients receiving these treatments. While the safety of SCIg in both PID and SID is well established, psychosocial factors and pre-existing comorbidities remain significantly understudied and insufficiently integrated into current risk assessments.

Pre-existing comorbidities did not influence the occurrence of LARs, with the exception of prior dermatological conditions. This association is biologically plausible, given that SCIg is administered directly into the skin, and pre-existing cutaneous disorders may predispose to LARs through mechanisms such as the Koebner phenomenon [28]. Future prospective studies incorporating serum cytokine profiling and local inflammatory markers could provide mechanistic insights into how pre-existing skin pathology may modulate local immune responses to SCIg, thereby complementing the clinical associations identified in our study. Conversely, employment status, social situation, mobility, independence, and self-care capacity were not associated with LARs, reinforcing the safety of SCIg even in elderly, frail, and comorbid patient populations. Additionally, as the vast majority of our cohort performed SCIg administration at home, factors such as waiting time before therapy, attending the clinic alone, or the time to patient release are unlikely to influence anxiety levels or LAR occurrence, in contrast to hospital-based IVIg. Nevertheless, mental health and subjective health perception emerged as significant determinants of LAR risk in our cohort. Specifically, the absence of anxiety or depression was associated with a substantially lower likelihood of LARs, indicating that patients reporting anxiety or depression may have a higher risk of experiencing local reactions. This association may be mediated by heightened pain perception, increased bodily hypervigilance, or underlying physiological mechanisms. These findings underscore the importance of systematically assessing psychological well-being prior to initiating SCIg therapy and considering early supportive interventions in patients reporting anxiety or depression. Additionally, greater distance to the healthcare center was independently associated with an increased frequency of LARs. This likely reflects reduced access to timely care and follow-up, a challenge particularly relevant in immunodeficiency reference units, which often serve patients from remote areas requiring specialized management. These findings align with previous evidence linking longer travel times or distances to healthcare facilities with poorer health outcomes, especially in complex or comorbid populations [29].

Interestingly, the exploratory multivariable model with the highest predictive performance identified four key variables with good discrimination and low risk for overfitting after internal validation, which can be graphically presented as a nomogram: pre-existing dermatological disease, abdominal perimeter, self-reported anxiety and/or depression, and distance to the hospital. By incorporating physiological, demographic, and psycho-emotional factors, this model offers a novel, multidimensional approach to risk stratification. These findings support the implementation of targeted preventive strategies—such as mental health support, enhanced patient education, teleconsultations for those living at greater distances, closer monitoring of underweight individuals, and optimized management of dermatological conditions—to reduce the risk of LARs in vulnerable subgroups. Importantly, these measures can help ensure that such patients are not excluded from SCIg therapy, thereby promoting more equitable access to care.

This multidimensional approach is particularly relevant for specialized nursing care, where comprehensive, individualized follow-up is essential. Nurses play a central role in patient education, monitoring, and the early identification of adverse reactions to SCIg. Notably, this study, designed, led, and conducted by nursing professionals, proposes a potential clinically applicable model for anticipating LARs and personalizing patient support from the outset of therapy.

Nevertheless, several limitations must be acknowledged. Firstly, this study’s retrospective design introduces a potential risk of recall bias, particularly for self-reported variables. Secondly, the predictive model has not yet undergone external validation, which will be essential prior to any routine clinical implementation. Moreover, despite the overall sample size, the inclusion of multiple multidimensional variables may have limited the statistical power, particularly in the context of multivariable analyses, potentially affecting the robustness of some associations. In addition, some variables—such as infusion site selection or needle length—may be jointly determined by patient anatomy and nursing preferences, introducing potential confounding. Given the observational nature of our study, the reported associations should not be interpreted as causal relationships; establishing causality would require prospective, long-term studies with standardized assessment of these factors. Furthermore, the absence of biological data precluded exploration of the underlying mechanisms driving the observed associations, an aspect that should be addressed in future research. The exclusion of pediatric patients limits the applicability of our findings to individuals aged 14 years and older. Although the multicenter design enhances the generalizability of the results, more than two-thirds of the patients were recruited from a single reference center, which may introduce some degree of center-related bias despite the use of standardized protocols and uniform data collection procedures. Finally, differences in staff training and administration practices across participating sites may have influenced both patient outcomes and the perception of SCIg therapy.

## 5. Conclusions

This multicenter, nursing-led study specifically explored risk factors associated with LARs in SCIg therapy based on real-world data in patients with PID and SID. By integrating physiological, dermatological, psychological, and geographical factors, we propose an exploratory model that could enable individualized risk stratification and support the implementation of tailored preventive strategies. Our findings emphasize the notable impact of pre-existing skin diseases on LAR occurrence, which may be further influenced by the location of injection sites and, potentially, by the frequency of administrations in relation to disease severity. These results underscore the need for a multidimensional approach in SCIg initiation and monitoring, particularly in vulnerable subgroups, to promote safer and more equitable access to immunoglobulin therapy. However, important limitations must be considered, including the retrospective design, the predominance of patients from a single reference center, and the lack of direct comparison with intravenous immunoglobulin in terms of secondary events. Building on these results, future prospective, multicenter studies should validate this model and assess whether early identification and management of key modifiable factors—such as anxiety and skin disease—can improve local tolerability and adherence. External validation, longitudinal follow-up, and incorporation of patient-reported outcomes will be essential to confirm these associations and facilitate their translation into routine clinical practice.

## Figures and Tables

**Table 1:** Summary of local and systemic adverse effects to subcutaneous immunoglobulin therapy.

Variable	*n*	Frequency (%)
**Local adverse effects**	163	98.19%
Local rash	108	65.06%
Local pain	53	31.93%
Pruritus	108	65.45%
Swelling	117	70.48%
Nodules	12	7.45%
Leak	18	10.84%
Hematoma	18	10.84%
Ulceration	4	2.41%
Necrosis	8	4.82%
**Systemic adverse effects**	67	40.36%
Headache	43	25.9%
Asthenia	29	17.47%
Fever	13	7.83%
Arthromyalgia	23	13.86%
Systemic rash	6	3.61%
Wear-off	33	24.63%

**Table 2:** Descriptive analysis of patient profiles according to SCIg cutaneous tolerability.

Variable	Category	No Local Adverse Effect	Local Adverse Effect	*p*-Value
Age		50.15 (SD 17.73)	46.01 (SD 18.74)	0.131
Diagnosis	PID	42 (70%)	103 (63.19%)	0.4288
	SID	18 (30%)	60 (36.81%)	
Sex	Men	24 (40%)	62 (38.04%)	0.8769
	Women	36 (60%)	101 (61.96%)	
Cytopenia	No	51 (85%)	147 (90.18%)	0.3378
	Yes	9 (15%)	16 (9.82%)	
Immune thrombopenia	No	54 (90%)	154 (94.48%)	0.2394
	Yes	6 (1%)	9 (5.52%)	
Anticoagulation	No	55 (91.67%)	152 (93.25%)	0.7704
	Yes	5 (8.33%)	11 (6.75%)	
Skin disease	No	53 (89.83%)	121 (74.69%)	0.0155 *
	Yes	6 (10.17%)	41 (25.31%)	
Hematopoietic stem cell transplantation	No	55 (93.22%)	146 (89.57%)	0.604
	Yes	4 (6.78%)	17 (10.43%)	
Solid organ transplantation	No	58 (98.31%)	150 (95.54%)	0.4514
	Yes	1 (1.69%)	7 (4.46%)	
Weekly SCIg dose		8.10 (SD 2.48)	8.02 (SD 2.93)	0.835
Total infusion volume (both 10% and 20%)		159.81 (SD 145.20)	155.88 (SD 123.05)	0.888
Posology	Every two weeks	7 (13.34%)	16 (9.82%)	0.0507
	Monthly	12 (20%)	28 (7.18%)	
	Other	1 (1.67%)	0 (0.0%)	
	Weekly	30 (50%)	65 (39.88%)	
	Every three weeks	9 (15%)	53 (32.52%)	
Injection points		1.93 (0.41)	1.84 (0.37)	0.124
Needle length	6 mm	0 (0%)	6 (3.7%)	0.023 *
	8 mm	2 (3.3%)	12 (7.4%)	
	9 mm	56 (93.3%)	120 (73.6%)	
	10 mm	1 (1.7%)	6 (3.7%)	
	12 mm	1 (1.7%)	19 (11.7%)	
SCIg formula	10%	23 (38.33%)	82 (50.31%)	0.1311
	20%	37 (61.67%)	81 (49.69%)	
Injection place	Abdomen	49 (81.67%)	88 (53.99%)	0.002 *
	Alternates abdomen and thighs	5 (8.33%)	44 (26.99%)	
	Alternates arms and thighs	1 (1.67%)	1 (0.61%)	
	Alternates abdomen, arms, and thighs	0 (0.0%)	3 (1.84%)	
	Arms	0 (0.0%)	2 (1.23%)	
	Thighs	5 (8.33%)	25 (15.34%)	
Needle thickness	26 G	37 (61.67%)	92 (56.44%)	0.5421
	27 G	23 (38.33%)	71 (43.56%)	
Infusion time (min)		80.75 (SD 74.88)	60.69 (27.17)	0.047 *
Abdominal perimeter		94.94 (SD 13.17)	87.34 (SD 12.57)	0.0002 *
Hospital distance (km)		20.76 (SD 28.63)	34.96 (SD 42.47)	0.005 *
Previous IVIg	No	19 (32.2%)	55 (33.74%)	0.8732
	Yes	40 (67.8%)	108 (66.26%)	
Laboral situation	Disability	8 (13.3%)	29 (17.8%)	0.085
	Employed	29 (48.3%)	59 (36.2%)	
	Unemployed	2 (3.3%)	8 (4.9%)	
	Student	3 (5%)	29 (17.8%)	
	Retired	18 (30%)	38 (23.3%)	
Familiar situation	Lives with a spouse of similar age	16 (27.12%)	54 (33.33%)	0.314
	Lives with family members without physical or mental dependency	31 (52.54%)	73 (45.06%)	
	Lives with family and/or spouse and shows some signs of dependency	8 (13.56%)	15 (9.26%)	
	Lives alone and has no children or they live far away	1 (1.69%)	13 (8.02%)	
	Lives alone and has children nearby	3 (05.08%)	7 (4.32%)	
Social situation	Active social relationships	57 (95%)	154 (94.48%)	0.334
	Social relationships only with family or neighbors	0 (0.0%)	5 (3.07%)	
	Social relationships both with family and neighbors	3 (5%)	4 (2.45%)	
Mobility and independence	I have no problems walking	43 (72.88%)	132 (80.98%)	
	I have some problems walking	13 (22.03%)	28 (17.18%)	
	I have to stay in bed	3 (5.08%)	3 (1.84%)	
Personal care	I have no problems with personal care	53 (89.83%)	147 (90.18%)	1
	I am unable to wash or dress myself	2 (3.39%)	6 (3.68%)	
	I have some problems washing or dressing myself	4 (6.78%)	10 (6.13%)	
Anxiety and depression	I am moderately anxious or depressed	2 (3.45%)	44 (27.5%)	2.87 × 10^−5^ *
	I am very anxious or depressed	0 (0.0%)	3 (1.88%)	
	I am neither anxious nor depressed	56 (96.55%)	113 (70.62%)	
Discomfort	I have no pain or discomfort	49 (84.48%)	97 (60.25%)	0.000519 *

G, gauge; IVIg, intravenous immunoglobulin; min, minutes; mm, millimeters; PID, primary immunodeficiency; SCIg, subcutaneous immunoglobulin; SID, secondary immunodeficiency; *, statistically significant

**Table 3:** Multivariate logistic regression model identifying independent predictors of local adverse reactions to SCIg.

Variable	Odds Ratio	Lower 95% CI	Upper 95% CI	*p*-Value
Abdominal perimeter	0.9550	0.9300	0.9800	<0.001
Previous skin disease	2.7500	1.0300	7.3800	0.044
Distance to hospital	1.0100	1.0000	1.0200	0.0504
Not feeling anxious or depressed	0.0892	0.0204	0.3900	0.001

## Data Availability

The raw data supporting the conclusions of this article will be made available by the authors on request.
